# Association between olanzapine concentration and metabolic dysfunction in drug-naive and chronic patients: similarities and differences

**DOI:** 10.1038/s41537-022-00211-5

**Published:** 2022-02-28

**Authors:** Dongyu Kang, Jinjun Lu, Wenqing Liu, Ping Shao, Renrong Wu

**Affiliations:** 1 Department of Psychiatry, and National Clinical Research Center for Mental Disorders, The Second Xiangya Hospital of Central South University, Changsha, 410011 Hunan China; 2 Department of Psychiatry, Jiangyin No.3 People’s hospital, Jiangyin, 214400 Jiangsu China; 3 Brain Hospital of Hunan Province, Changsha, 410000 Hunan China

**Keywords:** Psychiatric disorders, Schizophrenia

## Abstract

Second-generation antipsychotics are widely used to treat schizophrenia but their use could induce metabolic dysfunction. To balance efficacy and side effects, various guidelines recommend the use of therapeutic drug monitoring. Given the controversial relationship between olanzapine serum concentration and metabolic dysfunction, its use in clinical practice is still debated. To address this issue, we conducted a prospective cohort study to explore the associations in patients with schizophrenia. Specifically, first-episode drug-naive patients and patients with chronic schizophrenia were recruited. All participants received olanzapine monotherapy for 8 weeks. Anthropometric parameters and metabolic indices were tested at baseline and at week 8, and olanzapine serum concentration was tested at week 4. After 8 weeks of observation, body weight and BMI increased significantly in drug-naive patients. Moreover, triglycerides and LDL increased significantly in both drug-naive and chronic patients. Among chronic patients, those who have never used olanzapine/clozapine before had a significantly higher increase in weight and BMI than those who have previously used olanzapine/clozapine. Furthermore, olanzapine concentration was associated with changes in weight, BMI, and LDL levels in the drug-naive group and glucose, triglyceride and LDL levels in chronic patients who have not used olanzapine/clozapine previously. In conclusion, the metabolic dysfunction induced by olanzapine is more severe and dose-dependent in drug-naive patients but independent in patients with chronic schizophrenia. Future studies with a longer period of observation and a larger sample are warranted.

## Introduction

Schizophrenia, one of the top ten causes of disability worldwide, is a psychiatric disorder characterized by positive symptoms of hallucinations, delusions, disorganized speech, negative symptoms and cognitive deficits that affects nearly 1% of the world’s population^1^. Second-generation antipsychotics are widely used in treating patients with schizophrenia. Nevertheless, metabolic dysfunction, such as hyperglycaemia and hyperlipidaemia, induced by some antipsychotics, such as clozapine and olanzapine, causes serious concerns^2,3^. These side effects not only hamper treatment compliance but also cause severe medical morbidities, such as diabetes, cardiovascular disease, and even premature death^4–6^. Therefore, side effects have become a major concern in treatment selection for individuals with psychotic diseases. A recent network meta-analysis suggested that olanzapine, together with clozapine and amisulpride, was significantly more efficacious in alleviating overall symptoms than other antipsychotics^7^. However, olanzapine can cause severe metabolic disorders, such as weight gain, lipid and glucose metabolic dysfunction^7^. A previous meta-analysis indicated an average weight gain of 4.45 kg with clozapine and 4.15 kg with olanzapine during a 10-week course of antipsychotic treatment^8^. Mitchell et al. found that the rate of metabolic syndrome in patients with schizophrenia was 51.0% for clozapine and 28.2% for olanzapine in a more recent meta-analysis^9^. Komossa et al. compared olanzapine with other second-generation psychotics and found that olanzapine outperformed aripiprazole, quetiapine, risperidone and ziprasidone but not amisulpride and clozapine in improving general mental state; however, olanzapine induced more weight gain than other psychotics except for clozapine^10^. The mechanisms underlying these side effects, with restrictions and conflicting results, are still poorly understood.

Based on the assumption that there is a relationship between plasma drug concentrations and clinical effects, researchers have strived to determine the ideal “therapeutic window”, which is characterized by maximal effectiveness and maximal safety for antipsychotics^11^. In 2011, the Arbeitsgemeinschaft für Neuropsychopharmakologie und Pharmakopsychiatrie (AGNP) guideline strongly recommended the use of therapeutic drug monitoring of antipsychotic therapy because it allows the use of personalized pharmacotherapy by considering the interindividual variability of pharmacokinetics^12^. For olanzapine, the recommended reference range was 20–80 ng/ml, and the alert level was 150 ng/ml. When the AGNP guideline was updated in 2017, the alert level was lowered to 100 ng/ml, considering the high risk of post injection syndrome due to an excessively high concentration of olanzapine pamoate^13^. The American Psychiatric Association, in its latest guideline for schizophrenia, recommended the use of drug monitoring for clozapine in treatment-resistant patients with schizophrenia, suggesting that drug levels greater than 350 ng/ml should be used, if well tolerated, to achieve the highest level of efficacy^14^. Similar recommendations for clozapine have been made in the British Association of Psychopharmacology guideline as well^15^. Drug monitoring was recommended to evaluate antipsychotic medication adherence in acute treatment in Canadian Psychiatric Association guidelines for pharmacotherapy in patients with schizophrenia, as clinicians’ capacity to accurately identify those who are nonadherent is limited^16^. A recent study from Clinical Antipsychotic Trials of Intervention Effectiveness supported the recommendation by suggesting that antipsychotic blood level below the reference range was related to treatment failure, with 50.8% of the participants with drug level under reference range experiencing treatment failure^17^.

However, only a few studies have focused on the use of therapeutic drug monitoring for metabolic dysfunction induced by antipsychotics^18^. In 2005, Perry et al. reported a threshold olanzapine level of 20.6 ng/ml for significant weight gain during olanzapine treatment^19^. A recent study in first-episode patients with schizophrenia suggested that an olanzapine concentration > 23.28 ng/ml was a positive predictor of significant weight gain^20^. However, these conclusions remain controversial. A large sample study with approximately six hundred participants failed to determine any significant correlation^21^. Meanwhile, in high-dose olanzapine treatment, Kelly et al. also failed to determine an association between weight gain and olanzapine plasma levels^22^.

The time period of olanzapine-induced metabolic dysfunction has been reported. A previous study following 200 participants for 2 years indicated that the mean weight gain for olanzapine after 2 years was 10.2 kg, and the median treatment time before meeting the significant weight gain was 5 weeks^23^. Individuals receiving olanzapine treatment gained weight rapidly in the first 12 weeks of treatment (mean, 7.3 kg), the pace of weight gain slowed gradually, and the weight stabilized after a year (mean, 10.2 kg). Overall, the rate of weight gain could be best described as a bell-shaped binomial curve which raised then declined over time^23^. Interestingly, the therapeutic benefit was associated with weight gain side effects, and this phenomenon is most likely to be observed in olanzapine and clozapine treatment^24^.

The recently published (American Psychological Association) APA guideline for schizophrenia suggested that a ranking of antipsychotics or an algorithmic approach to pharmacological treatment selection, due to lack of clinical evidence, remains infeasible. Therefore, the clinician’s choice of a particular antipsychotic agent typically depends on its side effects^25^. The metabolic dysfunction of olanzapine may have a major potential effect on long-term mortality and thus limit its application^26^. Similar claims have been made by other guidelines as well^15,16^.

For treatment-resistant schizophrenia, defined by the Treatment Response and Resistance in Psychosis Working group as the persistence of significant symptoms despite adequate pharmacological treatment^27^, most of the clinical guidelines recommend clozapine as an appropriate treatment, while olanzapine appeared to have a modest advantage over other non-clozapine second-generation antipsychotics^15^. However, olanzapine and clozapine outstand with the most severe metabolic side effects after switching antipsychotics^28^. A recent meta-analysis conducted by Toby Pillinger et al. highlighted that a significant difference exists between antipsychotics in terms of metabolic side effects, with olanzapine and clozapine exhibiting the worst profile^29^. Moreover, there was no sufficient evidence for a conclusion on which is better for nonresponsive patients, such as adding dose, switching antipsychotic medication, or combining antipsychotic medications^30–32^. With these mixed conclusions, it has become rather important to explore novel antipsychotics or optimized treatment strategies, based on current available options, for a better prognosis in patients with schizophrenia.

Although therapeutic drug monitoring is recommended by guidelines to assure effectiveness and minimize the side effects of olanzapine^12^, the relationship between olanzapine concentration and metabolic dysfunction remains controversial. While the majority of those studies focused on patients with chronic schizophrenia, there is a lack of discussion in first-episode drug-naive patients^33^. Therefore, we conducted this prospective cohort study to fill this gap. We hypothesized that there is a difference between chronic and drug-naive patients with schizophrenia in the association of olanzapine concentration and metabolic dysfunction.

## Results

A total of 120 participants were assessed for eligibility. Of the 3 individuals excluded, 2 did not meet the inclusion criteria, and 1 declined to participate. In total, 117 patients were enroled. There were 51 participants assigned to the first-episode drug-naive group and 66 participants to the chronic group, all of which completed the 8-week observation (Supplement Fig. 1). Demographic and baseline outcome measurements were compared among the first-episode drug-naive group and all chronic patients (Table 1). The daily dose of olanzapine and 4-week serum concentration of olanzapine correlated significantly in all participants tested (*r* = 0.337, *p* < 0.001). The clinical symptoms, measured by positive and negative symptom scale (PANSS), improved significantly in both groups after olanzapine treatment, and no difference was found between the change in PANSS scores between the drug-naive group and the chronic group. Repeated-measures ANOVA indicated a significant time effect on PANSS scores (*F* 1,115 = 427.10, *p* < 0.001, *η*^2^ = 0.981) but no significant effect of time and group interaction (*F* 1,115 = 0.039, *p* = 0.844, *η*^2^ = 0.09) (Table 2).

**Table 1:** Compare demographics and baseline clinical outcomes between two groups^a^.

Baseline variables	Drug-naive group (*n* = 51)	Chronic group (*n* = 66)	*F*(1,116)/Chi Sq	*P* value
Demographic variables				
Gender (male, female)	37.3% (19, 32)	47.0% (31, 35)	1.109	0.292
Age (y)	27.50 (24.83 30.17)	38.82 (36.19 41.45)	35.350	<0.001
Smoking (yes, no)	11.8% (6, 45)	3.0% (2, 64)	3.445	0.077
Clinical variables				
Course (y)	0.66 (0.60 0.73)	10.80 (9.48 12.13)	179.885	<0.001
Olanzapine dose (mg)	18.63 (17.93 19.32)	19.13 (18.39 19.86)	3.209	0.334
Olanzapine Concentration (ng/ml)	63.14 (55.18 71.10)	74.87 (65.04 84.70)	3.209	0.076
Height (m)	1.63 (1.61 1.66)	1.64 (1.62 1.66)	0.317	0.575
Weight (kg)	55.91 (53.29 58.54)	62.64 (59.49 65.78)	9.971	0.002
BMI	20.86 (20.25 21.46)	23.13 (22.19 24.07)	14.446	<0.001
Fasting glucose (mmol/l)	4.56 (4.41 4.71)	4.95 (4.79 5.10)	12.282	0.001
Triglyceride (mmol/l)	1.24 (1.01 1.46)	1.23 (1.12 1.33)	0.008	0.119
Total cholesterol (mmol/l)	3.99 (3.74 4.24)	4.82 (4.57 5.06)	21.143	<0.001
LDL (mmol/l)	2.32 (2.14 2.51)	2.03 (1.86 2.20)	5.284	0.023
HDL (mmol/l)	1.22 (1.14 1.29)	1.22 (1.14 1.30)	0.019	0.892
PANSS	83.92 (79.97 87.87)	89.96 (87.17 92.56)	6.630	0.011

*BMI* body mass index, *LDL* low-density lipoprotein, *HDL* high-density lipoprotein, *PANSS* positive and negative symptom scale.

^a^ANOVA for continuous index and Chi-square or Fisher exact test for categorical index.

**Table 2:** Compare outcomes before and after treatment within two groups^a^.

	Drug-naive group (*n* = 51)					Chronic group (*n* = 66)					Repeated measurement		Change of outcomes	
	Week 0	Week 8	Δ^b^	F/U	*p*	Week 0	Week 8	Δ^b^	F/U	*p*	*F* value	P value	*p* value	*P* adj
Weight (kg)	55.91 (53.29 58.54)	58.92 (56.22 61.63)	2.99 (2.12 3.87)	922.500	0.017	62.64 (59.49 65.78)	62.82 (59.78 65.86)	0.18 (−0.73 1.10)	0.007	0.934	–	–	<0.001	0.035
BMI	20.86 (20.25 21.46)	21.98 (21.34 22.62)	1.14 (0.79 1.48)	6.579	0.012	23.13 (22.19 24.07)	23.22 (22.29 24.15)	0.09 (−0.25 0.44)	0.020	0.887	8.930	0.003	<0.001	0.054
Fasting glucose (mmol/l)	4.56 (4.41 4.71)	4.71 (4.56 4.85)	0.14 (0.02 0.26)	2.028	0.158	4.95 (4.79 5.10)	5.09 (4.89 5.30)	0.15 (−0.05 0.35)	1953.000	0.377	–	–	0.969	0.417
Triglyceride (mmol/l)	1.24 (1.01 1.46)	1.68 (1.41 1.96)	0.44 (0.22 0.67)	6.338	0.013	1.23 (1.12 1.33)	2.20 (1.88 2.51)	0.96 (0.66 1.26)	34.131	<0.001	3.315	0.071	0.011	0.004
Total cholesterol (mmol/l)	3.99 (3.74 4.24)	4.33 (4.05 4.62)	0.35 (0.13 0.56)	3.274	0.071	4.82 (4.57 5.06)	5.08 (4.80 5.36)	0.26 (0.02 0.50)	1825.500	0.181	–	–	0.628	0.944
LDL (mmol/l)	2.32 (2.14 2.51)	2.77 (2.52 3.03)	0.45 (0.24 0.66)	8.196	0.005	2.03 (1.86 2.20)	2.62 (2.41 2.83)	0.59 (0.40 0.78)	18.984	<0.001	3.019	0.085	0.330	0.557
HDL (mmol/l)	1.22 (1.14 1.29)	1.20 (1.13 1.27)	−0.01 (−0.06 0.05)	0.074	0.786	1.22 (1.14 1.30)	1.13 (1.04 1.22)	−0.63 (−0.15 0.03)	2.380	0.125	0.559	0.457	0.283	0.670
PANSS	83.92 (79.97 87.87)	54.66 (50.72 58.60)	−29.15 (−33.31 −24.99)	110.926	<0.001	89.96 (87.17 92.56)	61.23 (58.36 64.10)	−28.60 (−32.36 −24.83)	211.389	<0.001	9.908	0.002	0.844	0.307

*LDL* low-density lipoprotein, *HDL* high-density lipoprotein, *PANSS* positive and negative symptom scale, *BMI* body mass index.

^a^To compare baseline and endpoint within each group, ANOVA was conducted for continues variables. Mann–Whitney test was conducted if not normal distributed. To compare the change of outcomes between two groups, the General linear regression random effect model with course, smoking, age adjusted. Repeated Measurement analysis was conducted to compare the change of outcomes between two groups over time.

^b^The change between endpoint and baseline.

### Demographic and baseline outcome measurements

There were 19 males and 32 females in the drug-naive group and 31 males and 35 females in the chronic group. In the metabolic risk^-^ subgroup, 8 participants used risperidone, 9 participants used aripiprazole, and 2 used amisulpride, while in the metabolic risk^+^ subgroup, 6 participants used clozapine, and 41 participants used olanzapine before enrolment. There was no significant difference in sex, smoking history, after-enrolment olanzapine daily dosage, serum olanzapine concentration or body height between the two groups at baseline. Naturally, the age of the chronic group (mean = 27.50, 95% CI, 24.83–30.17) was significantly higher than that of the drug-naive group (mean = 38.82, 95% CI, 36.19–41.45) (*η*^2^ = 0.237, *p* < 0.001). The chronic group had worse metabolic conditions at baseline. Specifically, body weight (*F* 1,116 = 9.97, *p* = 0.002, *η*^2^ = 0.08), BMI (*F* 1,116 = 14.45, *p* < 0.001, *η*^2^ = 0.112), fasting glucose (*F* 1,116 = 12.28, *p* = 0.001, *η*^2^ = 0.097), total cholesterol (*F* 1,116 = 21.14, *p* < 0.001, *η*^2^ = 0.159) and low-density lipoprotein (LDL) (*F* 1,116 = 5.284, *p* = 0.023, *η*^2^ = 0.045) of the chronic group were significantly higher than those of the drug-naive group. There was no significant difference in high-density lipoprotein (HDL) or triglycerides between the two groups at baseline. The three group analysis between the drug-naive group and the Metabolic Risk^-^ and Metabolic Risk^+^ subgroups displayed similar results (Supplement Table 6).

### Changes in body weight and BMI

After 8 weeks of olanzapine monotherapy, BMI (mean = 1.14, 95% CI, 0.79–1.48, *F* 1,100 = 922.50, *p* = 0.012, *η*^2^ = 0.062) and body weight (mean = 2.99, 95% CI, 2.12–3.87, *U* = 922.50, *p* = 0.017, *η*^2^ = 0.025) increased significantly in the drug-naive group compared with baseline. In contrast, in the chronic group, neither body weight nor BMI showed a significant increase compared to baseline (Table 2).

As presented in Table 2, the drug-naive group had a conspicuous increase in body weight (mean = 2.99, 95% CI, 2.12–3.87), which was significantly higher than that in the chronic group (mean = 0.18, 95% CI, −0.73 to 1.10) (*F* 1,115 = 18.80, *p* < 0.001, *η*^2^ = 0.142). So was the increase in BMI in the drug-naive group (Mean=1.14, 95% CI, 0.79 to 1.48), which was significantly higher than that in the chronic group (mean = 0.09, 95% CI, −0.25 to 0.44) (*F* 1,115 = 17.571, *p* < 0.001, *η*^2^ = 0.134). Repeated-measures ANOVA on BMI suggested a significant interaction between time and group on BMI (*F* 1,115 = 17.57, *p* < 0.001, *η*^2^ = 0.134) and a significant difference between the two groups (*F* 1,115 = 8.93, *p* = 0.003, *η*^2^ = 0.073). The subgroup analysis, comparing three groups after adjustment for disease course, smoking history, and age, indicated that the metabolic risk^-^ subgroup (mean = 2.11, 95% CI, 0.62–3.59) and drug-naive group (mean = 2.99, 95% CI, 2.12–3.87) had a significantly higher increase in body weight than the metabolic risk+ subgroup (mean = −0.60, 95% CI, −1.68, 0.49) (*F* 2,114 = 14.45, *p* < 0.001, *η*^2^ = 0.116). The metabolic risk^-^ subgroup (mean= 0.80, 95% CI, 0.24–1.37) and drug-naive group (mean = 1.14, 95% CI, 0.79–1.48) also had a significantly higher BMI increase compared with metabolic risk^+^ subgroup (mean = −0.19, 95% CI, −0.61 to 0.22) (*F* 2,114 = 13.39, *p* < 0.001, *η*^2^ = 0.106). Meanwhile, no significant difference was found in the change in body weight or BMI between the drug-naive group and metabolic risk^-^ subgroup (Table 3). Similar results were found when we compared the difference between the two subgroups of chronic patients (Supplement Table 7).

**Table 3:** Three group analysis of changes of clinical outcomes^a^.

	Drug-naive group (*n* = 51)	Metabolic risk^+^ group (*n* = 47)	Metabolic risk^−^ group (*n* = 19)	*F*(2, 114)	*p* value	*p* adj	D vs R^+^	D vs R^−^	R^−^ vs R^+^
Weight (kg)	2.99 (2.12 3.87)	−0.60 (−1.68 0.49)	2.11 (0.62 3.59)	14.452	<0.001	0.001	(2.24 4.94)**	(−0.90 2.68)	(0.90 4.50)**
BMI	1.14 (0.79 1.48)	−0.19 (−0.61 0.22)	0.80 (0.24 1.37)	13.391	<0.001	0.002	(0.81 1.85)*	(−0.35 1.02)	(0.30 1.69)**
Fasting glucose (mmol/l)	0.14 (0.02 0.26)	0.22 (−0.02 0.45)	−0.02 (−0.44 0.40)	0.817	0.445	–	–	–	–
Triglyceride (mmol/l)	0.44 (0.22 0.67)	0.96 (0.56 1.37)	0.96 (0.58 1.34)	3.341	0.039	0.015	(−0.95 −0.09)**	(−1.08 0.05)*	(−0.58 0.57)
Total cholesterol (mmol/l)	0.35 (0.13 0.56)	0.26 (−0.02 0.53)	0.28 (−0.26 0.82)	0.121	0.886	–	–	–	–
LDL (mmol/l)	0.45 (0.24 0.66)	0.62 (0.38 0.85)	0.52 (0.19 0.86)	0.573	0.566	–	–	–	–
HDL (mmol/l)	−0.01 (−0.06 0.05)	−0.08 (−0.18 0.02)	−0.01 (−0.21 0.20)	0.993	0.374	–	–	–	–
PANSS	−29.15 (−33.31 −24.99)	−30.83 (−35.58 −26.08)	−22.88 (−28.21 −17.54)	1.879	0.158	–	–	–	–

*D* Drug naive group, *R*^*+*^ Olanzapine or clozapine used group, *R*^−^ Olanzapine or clozapine-naive group, *LDL* low-density lipoprotein, *HDL* high-density lipoprotein, *PANSS* positive and negative symptom scale, *BMI* body mass index.

*p < 0.05; **p < 0.01.

^a^ANOVA was used for three group analysis, LSD was conducted for post hoc analysis. For the statistically significant result, general linear regression random effect model was conducted with course, smoking, age adjusted.

Spearman correlation analysis of all participants indicated that antipsychotic-related metabolic risk was significantly associated with changes in body weight (*r* = −0.479, *p* < 0.01), change of BMI (*r* = −0.463, *p* < 0.01) and change rate of BMI (*r* = −0.482, *p* < 0.01) after 8 weeks of olanzapine monotherapy (Supplement Table 1). In the drug-naive group, the serum concentration of olanzapine was correlated with changes in body weight (*r* = 0.375, *p* < 0.01), BMI (*r* = 0.365, *p* < 0.01), and the change rate of BMI (*r* = 0.324, *p* < 0.05) (Supplement Table 2). However, this phenomenon was not found in the chronic group (Supplement Table 3) or in the metabolic risk^-^ (Supplement Table 4) or metabolic risk + (Supplement Table 5) subgroups.

### Changes in glucose and lipid metabolism

In the drug-naive group, triglyceride (mean = 0.44, 95% CI, 0.22–0.67, F 1,100 = 6.34, *p* = 0.013, *η*^2^ = 0.06) and LDL (mean = 0.45, 95% CI, 0.24–0.66, *F* 1,100 = 8.20, *p* = 0.005, *η*^2^ = 0.077) levels increased significantly after 8 weeks of treatment, while total cholesterol, fasting glucose and HDL levels did not change. Similarly, in the chronic group, triglyceride (mean = 0.96, 95% CI, 0.66–1.26, *F* 1,130 = 34.13, *p* < 0.001, *η*^2^ = 0.209) and LDL (mean = 0.59, 95% CI, 0.40–0.78, *F* 1,130 = 18.98, *p* < 0.001, *η*^2^ = 0.129) levels increased significantly after treatment, while fasting glucose, total cholesterol, and HDL levels did not (Table 2).

The increase in triglyceride levels in the drug-naive group (Mean=0.44, 95% CI, 0.22–0.67) was significantly lower than that in the chronic group (mean = 0.96, 95% CI, 0.66–1.26) (*F* 1,115 = 6.74, *p* = 0.011, *η*^2^ = 0.056). However, the changes in fasting glucose, total cholesterol, LDL, and HDL were not significantly different between the two groups (Table 2). The subgroup analysis showed similar results, indicating a steeper triglyceride increase in the chronic group, regardless of whether olanzapine/clozapine had been used before (Table 3 and Supplement Table 7). This evidence suggested a potentially more severe olanzapine-induced impairment of triglyceride metabolism in chronic patients with schizophrenia.

When taking all participants into account, the serum olanzapine concentration was positively correlated with the change in fasting glucose level (*r* = 0.194, *p* < 0.05). A similar result was found in the metabolic risk^-^ subgroup, in which the serum olanzapine concentration was positively correlated with the change in fasting glucose (*r* = 0.531, *p* < 0.05), but not in the metabolic risk^+^ subgroup. Interestingly, the serum olanzapine concentration was positively correlated with LDL levels in all participants (*r* = 0.275, *p* < 0.01), the drug-naive group (*r* = 0.472, *p* < 0.01), and the metabolic risk^-^ subgroup (*r* = 0.492, *p* < 0.01) but not in the metabolic risk^+^ subgroup, which indicated a potential acute dose-dependent impairment of glucolipid metabolism at the first exposure to olanzapine (Supplement Tables 1–5). In addition, olanzapine concentration was positively correlated with the change in total cholesterol level (*r* = 0.313, *p* < 0.05) in the drug-naive group (Supplement Table 2). The results were controversial with regard to triglyceride levels. Olanzapine concentration was positively correlated with the change in triglyceride levels in the metabolic risk^-^ subgroup (*r* = 0.472, *p* < 0.05) but negatively correlated with the change in triglyceride levels in the metabolic risk^+^ subgroup (*r* = −0.312, *p* < 0.05) and was not significantly associated in the chronic group (Supplement Tables 3, 4 & 5).

### Association between olanzapine level and metabolic indices

**Fig. 1:**
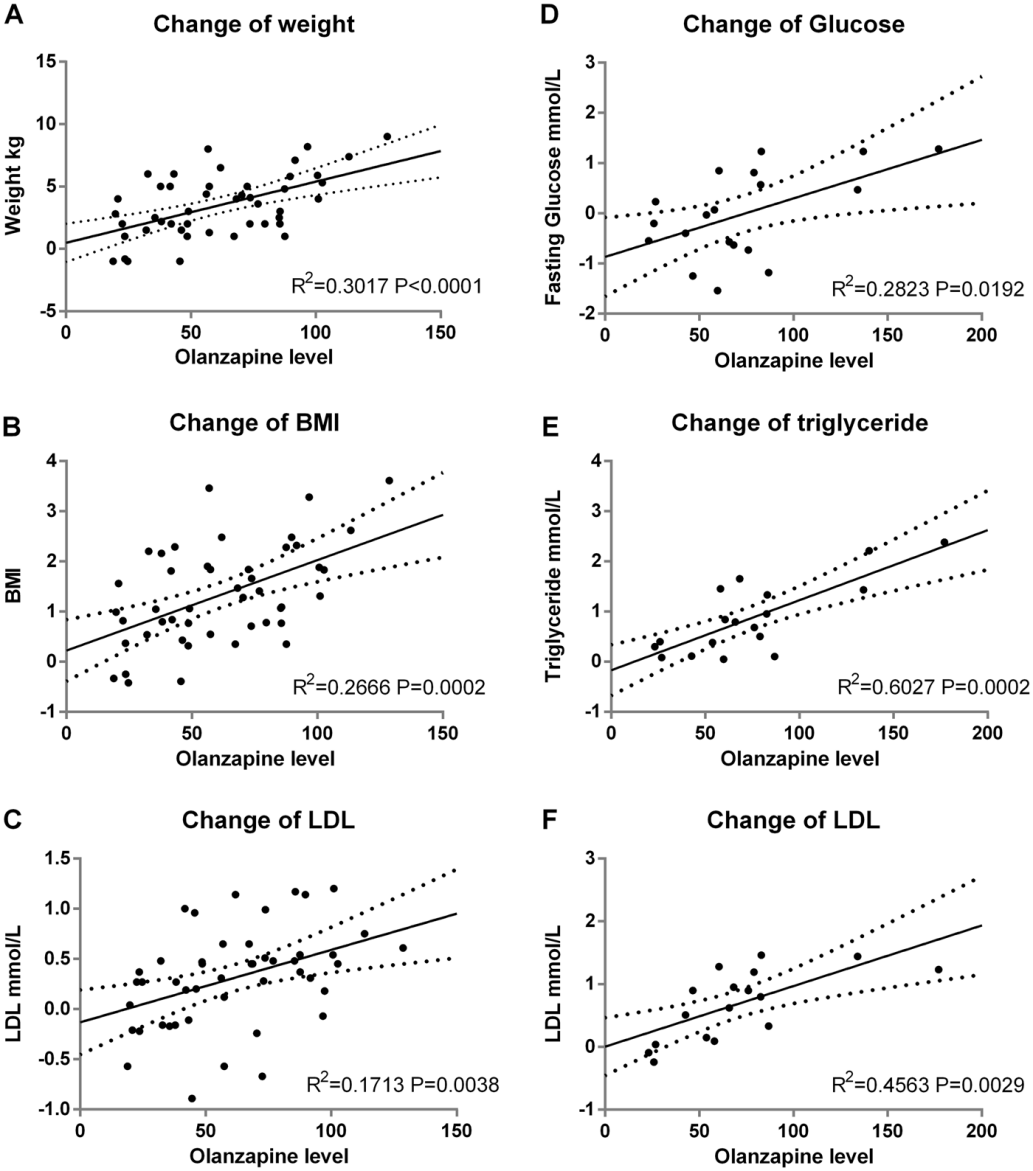
General linear regression analysis for olanzapine concentration. Changes of metabolic measurements with olanzapine levels (ng/ml) in drug-naive group and metabolic risk^−^ subgroup. The error bars are showed as dotted line. BMI body mass index, LDL low-density lipoprotein. **A**, **B** and **C** General linear analysis was conducted in drug-naive group. **D**, **E** and **F** General linear analysis was conducted in metabolic risk^−^ subgroup.

General linear regression analysis was conducted for those significant correlation results found above with a few outliers excluded, which did not change the result, and the results are visualized in Fig. 1. In the drug-naive group, the change in weight, BMI, and LDL could be predicted by the olanzapine serum level. Meanwhile, in metabolic risk^-^ subgroup, glucose, triglycerides, and LDL could be predicted by olanzapine serum levels. However, those results were not found in the chronic metabolic risk^+^ subgroup, providing evidence for a potentially different metabolic side-effect pattern for olanzapine-naive patients and patients who used olanzapine previously.

Furthermore, we conducted multiple linear regression analysis for potential predictive factors in each group. In the drug-naive group, serum olanzapine concentration in week 4 could predict the change in BMI (*β* = 0.354, *p* = 0.019) after the 8-week treatment, after adjustment for smoking history, course of disease, and sex. Similarly, the serum olanzapine concentration at week 4 could predict the change in body weight (*β* = 0.376, *p* = 0.013) with the model adjusted on the boundary of significance (*p* = 0.056). However, this phenomenon was not found in chronic patients (Table 4). In the drug-naive group, the serum level of olanzapine could predict the change in LDL after treatment (*β* = 0.514, *p* = 0.001), adjusted for smoking history, disease course and sex difference (*p* = 0.011). In metabolic risk^−^ subgroup, serum olanzapine levels could predict the change in triglycerides (*β* = 0.704, *p* = 0.021), with age also included in the model (*β* = 0.604, *p* = 0.028). In addition, serum olanzapine levels predicted the change in fasting glucose (*β* = 0.984, *p* = 0.002) after adjustment for age and sex. However, those phenomena were not found in the chronic metabolic risk^+^ subgroup.

**Table 4:** Multiple Linear regression analysis of outcomes^a^.

**Drug naive group**	**Ola concentration**		**Smoke**		**Course**		**Gender**		**Model summary**	
	***β***	***p***	***β***	***p***	***β***	***p***	***β***	***p***	**Adjusted R square**	***P*** **value**
Weight (kg)	0.376	0.013	0.130	0.365	0.067	0.631	0.065	0.637	0.109	0.056
BMI	0.354	0.019	0.164	0.254	0.096	0.491	0.119	0.387	0.118	0.046
LDL (mmol/L)	0.514	0.001	−0.138	0.320	0.004	0.975	0.119	0.366	0.179	0.011
**Metabolic risk**^**−**^ **group**	**Ola concentration**		**Course**		**Age**		**Gender**		**Model summary**	
	***β***	***p***	***β***	***p***	***β***	***p***	***β***	***p***	**Adjusted R square**	***P*** **value**
Fasting glucose (mmol/L)	0.984	0.002	–	–	0.604	0.028	0.297	0.161	0.396	0.014
Triglyceride (mmol/L)	0.704	0.021	0.036	0.893	–	–	0.322	0.157	0.336	0.027
LDL (mmol/L)	0.186	0.585	−0.120	0.717	–	–	−0.278	0.309	0.007	0.403

*Ola* Olanzapine, *BMI* body mass index, *LDL* low-density lipoprotein.

^a^For measures not normal distributed, multiple linear regression analysis was conducted after ranking.

### Exploring the possible reason for difference

There was a significantly higher increase in both weight gain and BMI in the group of drug-naive patients after the follow-up. As mentioned above, the speed of weight gain decreases over time, and eventually, the weight becomes stable. Intuitively, we might assume that for chronic patients, the speed of weight gain has slowed down compared to first-episode patients. Therefore, the change in weight or BMI should be correlated with the baseline weight or BMI, indicating the process of antipsychotic-induced weight gain. As expected, the multilinear regression analysis suggested that the baseline weight/BMI and previous medication could predict the change in weight/BMI in our data (model summary, *R*^2^ = 0.196, *p* < 0.001; *R*^2^ = 0.187, *p* < 0.001), suggesting less weight gain if the patient had a higher body weight/BMI (*β* = −0.222, *p* = 0.015; *β* = −0.231, *p* = 0.015) or had taken olanzapine/clozapine before (*β* = −0.391, *p* = 0.001). Other possible factors, including age, course of the disease, medication history and psychotic syndrome (PANSS score), were adjusted (Supplemental Tables 8 and 9).

Our data suggested a more severe impairment of triglyceride metabolism induced by olanzapine in the chronic patient group, while the change of triglyceride is related to olanzapine concentration. However, this phenomenon was not found in drug-naive patients. When involving all participants, the change in triglycerides was related to the baseline fasting glucose level (*r* = 0.218, *p* < 0.05) and baseline cholesterol level (*r* = 0.199, *p* < 0.05). Nonetheless, the baseline levels of cholesterol and glucose failed to predict the change in triglycerides in the multilinear regression analysis (*R*^2^ = 0.04, *p* = 0.058). Further cohort research enroling weight/BMI matched participants may provide more evidence on the difference of metabolic dysfunction induced by olanzapine in first-episode and chronic patients (Supplement Tables 10 and 11).

## Discussion

The main findings of the present study are the significant associations between metabolic dysfunction and olanzapine serum levels in drug-naive or olanzapine/clozapine-naive patients. However, in chronic patients who previously used olanzapine, the reuse of olanzapine caused severe dyslipidaemia in a dose-independent manner. After 8 weeks of olanzapine monotherapy, body weight and BMI increased significantly in drug-naive patients but not in chronic patients. Meanwhile, triglycerides and LDL increased significantly in both drug-naive patients and chronic patients. We then divided the chronic patients into two subgroups and found that the metabolic risk^-^ patients, similar to drug-naive patients, were more likely to experience severe side effects than the metabolic risk^+^ patients, as they had a significantly higher increase in weight and BMI. Moreover, the Spearman correlation test and linear regression analysis were conducted, indicating that body weight, BMI and LDL levels in drug-naive patients and glucose, triglyceride and LDL levels in chronic olanzapine^-^ patients were correlated with serum olanzapine concentrations. In conclusion, the use of olanzapine-induced significant metabolic dysfunction in drug-naive patients with schizophrenia in a dose-dependent manner. For chronic patients, the use of olanzapine could induce serious dyslipidaemia in a dose-independent manner. We assume that the previous use of antipsychotics could alter the vulnerability to antipsychotic-induced metabolic dysfunction when exposed again. Further cohort studies on whether previous antipsychotic treatment could predict metabolic dysfunction in later pharmacotherapy could be promising.

Our result in chronic patients is similar to a recent study conducted in Taipei, including 151 chronic patients with schizophrenia using a stable dose of olanzapine for at least three months, which failed to determine a significant correlation between olanzapine level and weight change; nevertheless, the *N*-desmethyl-olanzapine (DMO) concentration-to-dose ratio was negatively correlated with weight, BMI, and waist circumference^34^. For first psychosis episode patients, a previous study found a positive correlation of olanzapine level with weight gain, which was consistent with our findings^20^. One retrospective study that included 39 acutely ill patients reported that olanzapine concentrations above 20.6 ng/ml were associated with significant weight gain^19^. However, Zabala et al. reported a negative result in a pilot study, as olanzapine concentration does not seem to be a reliable indicator for early drug effect and adverse effects in first-episode patients^33^. Several other studies have found no significant relationship between olanzapine levels and weight gain^21,22,35^, and one possible explanation is that all those studies enroled chronic patients, whose side effects could be dose-independent, according to our data.

Recent meta-analyses and real-world studies have suggested that olanzapine and clozapine show small but significant differences in measures of overall efficacy compared to other oral antipsychotics^7,36–38^. Nevertheless, olanzapine and clozapine have the worst metabolic side-effect profiles^29,38^. Since the APA guideline in 2021 suggested that it is not possible to have an evidence-based ranking of antipsychotic selection for significant heterogeneity and limitation in currently published data, the medication’s side-effect profile has been a significant factor when choosing medication with patients and their caregivers^14^. Therefore, a thorough evaluation and discussion with patients and their caregivers on the benefits and risks of olanzapine are recommended because the level of efficacy almost overlaps with the level of weight gain.

Moreover, TDM is more important if olanzapine and its metabolites were taken together. The metabolism of olanzapine mainly depends on CYP (cytochrome P450) 1A2 and CYP2D6 for oral intake and long-term injection administration^13,39^. The pharmacokinetics of olanzapine vary between individuals. Factors such as sex, age, comedication, smoking and inducers of P450 enzymes could have significant effects^40^. The main circulating metabolites are 10-N-glucuronide and 4′-N-DMO^41,42^. In Lu’s studies, the ratio of olanzapine level to DMO level was significantly negatively correlated with the PANSS score, and the ratio of DMO level to olanzapine dose was negatively correlated with metabolic measurements, proposing an optimal ratio of olanzapine level to DMO level between 3 and 6 for the balance of efficacy and metabolic side effects^34,43^. DMO has been reported to be negatively correlated with metabolic dysfunction in several other studies, suggesting a potential inverse effect to the main compound olanzapine^35,44,45^. Interestingly, DMO administration in high-fat diet-induced obese mice significantly reduced body weight and fat mass, accompanied by morphological changes in white adipose tissue^46^. Further studies on the so-called “metabolic normalization role” of DMO should be promising.

In our data, 4 participants had olanzapine levels lower than 20 ng/ml, 65 participants were within the recommended range 20–80 ng/ml as mentioned above, and 34 participants had olanzapine levels higher than 80 ng/ml. The change in PANSS score was not different between patients with the recommended level of olanzapine and those with a higher level, supporting the proposal that a higher dose of olanzapine was not related to better efficacy^47^. For all participants whose olanzapine level was between 20–80 ng/ml (*n* = 74), the drug level was related to the change in LDL (*r* = 0.268, *p* = 0.022). For drug-naive participants with a drug level within the recommended range (*n* = 34), the level was still related to the change in LDL (*r* = 0.355, *p* = 0.039) but not to body weight or BMI. For metabolic risk^-^ participants with recommended levels (*n* = 13), the drug level was also related to the change in LDL (*r* = 0.685, *p* = 0.01) but not to glucose and triglycerides. Nevertheless, for metabolic risk^+^ participants with recommended drug levels, the level was not related to any metabolic measurements. Similar to the results above, there was a consistent relationship between olanzapine levels and changes in LDL in different types of participants who never used olanzapine/clozapine previously. However, future studies with larger sample sizes are needed to draw a conclusion.

In our study, the PANSS score was significantly improved in both the drug-naive patient group and the chronic patient group, without a significant difference between the two groups. Intuitively, clinicians would assume that the efficacy of antipsychotics would be poorer in relapsed patients with chronic schizophrenia. Indeed, Takeuchi et al. reported significant episode-by-time interactions for antipsychotic treatment response in favour of the first episode compared to the second episode^48^. However, we did not find any significant difference between drug-naive patients and chronic patients. This might be due to the limited sample size and observation period.

To the best of our knowledge, few studies have investigated the relationship between metabolic dysfunction induced by olanzapine and drug serum concentration in drug-naive patients with schizophrenia^19,20,33^^,^ and no study has compared this relationship in both drug-naive patients and chronic patients before. Our results indicated the major difference between drug-naive patients and chronic patients in the relationship between metabolic dysfunction and drug concentration. This study has some limitations. First, we did not test the serum level of DMO in this study. Second, the tests were only performed at baseline and at week 8. This design may omit the dynamic of changes in metabolic measures and olanzapine concentration. Third, the sample size was limited, and patients from only two centres were enroled, which may have resulted in homogenous sampling. Future studies monitoring metabolic changes and pharmacokinetics with multicentre designs in larger populations are warranted.

Despite limitations, our study provides valuable evidence suggesting that olanzapine-induced metabolic dysfunction was olanzapine dose-dependent in drug-naive patients but independent in patients who used olanzapine/clozapine before. Metabolic dysfunction induced by olanzapine is a major concern for its clinical application. We suggest that when changing the medication to olanzapine, drug concentration monitoring is necessary for olanzapine-naive patients, and a thorough evaluation of the benefits and risks of olanzapine is required for chronic patients.

## Methods

### Trial design

This study was a prospective cohort trial designed to evaluate the relationship between single-time olanzapine serum concentration and metabolic dysfunction induced by olanzapine in first-episode drug-naive and chronic patients. The recruited patients were aged 18–45 years and met the Diagnostic and Statistical Manual of Mental Disorders (DSM-V) diagnostic criteria of schizophrenia. The study took place at the inpatient Department of Psychiatry of the Second Xiangya Hospital at Central South University in Hunan and the Department of Psychiatry of Jiangyin No.3 People’s Hospital in Jiangsu. The ethics committee of the Second Xiangya Hospital of Central South University approved this trial protocol. All participants who had full mental capacity or their caregivers provided written informed consent. Clinical trial registration: NCT03451734. This research has been a part of a multicentre clinical trial, and its protocol has been published elsewhere^49^. This study followed the Strengthening the Reporting of Observational Studies in Epidemiology (STROBE) reporting guidelines.

### Participants

Participants whose clinicians and caregivers had already decided to initiate olanzapine treatment or change to olanzapine treatment were recruited from March 2018 to September 2020. For first-episode patients, the inclusion criteria included the first psychotic episode of schizophrenia, having not taken any antipsychotics before, and having a disease course of less than a year. For chronic patients, the inclusion criteria included use of a stable dose of antipsychotics for at least 3 months but not having a full clinical recovery and willing to receive olanzapine monotherapy recommended independently by clinicians, with a disease course of more than 5 years. Other shared inclusion criteria included participants age 18–45 years and met the diagnostic criteria of the DSM-V. The exclusion criteria were as follows: (1) female patients who were pregnant or lactating at the time of enrolment, (2) patients with mental retardation or addictive disorders, and (3) patients with specific systemic diseases, such as cardiovascular diseases and hypertension. In the following subgroup analysis, investigators divided the chronic patients into the metabolic risk^+^ subgroup (chronic patients who had previously used olanzapine/clozapine) and the Metabolic Risk^-^ subgroup (chronic patients who had never used olanzapine/clozapine) according to their previous medication history. Written informed consent was provided by all participants or parents or statutory guardians as required.

### Outcome measures

Primary outcomes were serum olanzapine concentration, change in body weight, and body mass index (BMI, calculated as the ratio of weight in kilograms divided by height in metres squared). Secondary outcomes were the increase in the levels of lipids, which included HDL, low-density lipoprotein cholesterol (LDL), total cholesterol, and triglycerides. Psychopathologic symptoms were measured by PANSS with an independent assessor.

### Data collection

All participants received olanzapine monotherapy and were scheduled to have a clinical evaluation at baseline and at week 8. For drug-naive patients, olanzapine medication was initiated at 5 mg per day and titrated to the minimum effective dose within two weeks. The dose of olanzapine ranged from 10 to 20 mg per day in all participants. For chronic patients, the original medication was switched to olanzapine in a cross titration approach within two weeks^50^. None of the participants received other antipsychotics, hormonal contraceptives or drugs with metabolic effects after enrolment. All medications were administered at 11 a.m. and 5 p.m. each day. Fasting serum olanzapine concentration was tested at 7 a.m. at the end of week 4 using Agilent 1260 high-performance liquid chromatography^51^ with an average recovery rate of 96.61% of olanzapine in our study. The baseline assessments included demographics, medical history, physical examination, anthropometric measurements (weight and height), and PANSS score. The baseline laboratory tests included fasting glucose, lipid levels, liver and renal function, blood counts, and electrocardiograms. At week 8, all baseline clinical assessments and laboratory tests were repeated (Supplement Fig. 1).

### Statistical analysis

The data analysis was conducted in a blinded manner from October 2020 to March 2021. Statistics are presented as the mean and confidence interval for continuous variables and as frequencies for categorical variables. Between-group comparisons at baseline and within-group comparisons between baseline and endpoint were analyzed using one-way ANOVA or Mann–Whitney *U* test for continuous variables and chi-squared or Fisher’s exact test for categorical variables. Repeated-measures analysis was used to compare group differences over time. To compare the changes in outcomes between the two groups, we used the Kruskal–Wallis test and general linear regression random effect model adjusted for disease course, smoking history, and age. For further analysis, we divided the chronic patients into two subgroups, the metabolic risk^+^ subgroup and the metabolic risk^-^ subgroup. *A post hoc* test was conducted with LSD (least significant difference) to adjust the three-group analysis. The Spearman correlation test was conducted in all participants and within each subgroup, examining potential monotonic associations between variables. The antipsychotic-related metabolic risk (drug-naive, olanzapine/clozapine-naive, olanzapine/clozapine used) was considered a variable in the correlation test. General linear regression and multiple linear regression tests were then used to reveal potential predictors for metabolic side effects induced by olanzapine. The results were considered statistically significant if a two-tailed *P value* was <0.05. All analyses were conducted by using the Statistical Package for Social Sciences, version 23 (SPSS Inc, Chicago, Illinois).

## Supplementary information

Supplement tables

Supplement Figure

CONSORT chechlist

STROBE checklist

## Data Availability

The data will be available only to whose proposition on use of the data, for scientific research specified in a proposal, has been approved by corresponding author [RW].
