# Supplementary material for: Association between olanzapine concentration and metabolic dysfunction in drug-naive and chronic patients: similarities and differences
Source: Schizophrenia (Heidelb). 2022 Feb 28;8(1):9. doi: 10.1038/s41537-022-00211-5 (PMC8885747; doi:10.1038/s41537-022-00211-5)
Supplement: Supplementary file 1 — Supplement tables [file 41537_2022_211_MOESM1_ESM.docx]

Supplement Table 1 the Spearman relation test in all participants

| All Participants | Gender | Smoke | Metabolic Risk | Course(y) | Olanzapine Concentration | weight | BMI | BMI rate | FG | TG | TC | LDL | HDL | PANSS |
| --- | --- | --- | --- | --- | --- | --- | --- | --- | --- | --- | --- | --- | --- | --- |
| Gender | 1.000 | .029 | -.071 | -.128 | .089 | .078 | .121 | .139 | .069 | -.132 | .075 | .039 | .089 | -.083 |
| Smoke | .029 | 1.000 | -.139 | -.145 | .225* | .152 | .153 | .137 | .168 | -.236* | .095 | -.006 | .014 | -.146 |
| Metabolic Risk | -.071 | -.139 | 1.000 | .782** | .110 | -.479** | -.463** | -.482** | -.010 | .129 | -.085 | .152 | -.128 | -.032 |
| Course(y) | -.128 | -.145 | .782** | 1.000 | .008 | -.356** | -.340** | -.382** | -.063 | .073 | -.087 | .120 | -.108 | .055 |
| Olanzapine Concentration | .089 | .225* | .110 | .008 | 1.000 | -.005 | -.006 | -.019 | .194* | .001 | -.039 | .275** | -.112 | -.083 |
| Weight | .078 | .152 | -.479** | -.356** | -.005 | 1.000 | .994** | .988** | .045 | -.078 | .254** | .063 | .412** | .076 |
| BMI | .121 | .153 | -.463** | -.340** | -.006 | .994** | 1.000 | .992** | .048 | -.089 | .249** | .071 | .422** | .075 |
| BMI rate | .139 | .137 | -.482** | -.382** | -.019 | .988** | .992** | 1.000 | .032 | -.098 | .249** | .067 | .446** | .065 |
| FG | .069 | .168 | -.010 | -.063 | .194* | .045 | .048 | .032 | 1.000 | .120 | .199* | .059 | .023 | -.181 |
| TG | -.132 | -.236* | .129 | .073 | .001 | -.078 | -.089 | -.098 | .120 | 1.000 | .137 | .178 | -.091 | -.031 |
| TC | .075 | .095 | -.085 | -.087 | -.039 | .254** | .249** | .249** | .199* | .137 | 1.000 | .301** | .599** | .049 |
| LDL | .039 | -.006 | .152 | .120 | .275** | .063 | .071 | .067 | .059 | .178 | .301** | 1.000 | .200* | .112 |
| HDL | .089 | .014 | -.128 | -.108 | -.112 | .412** | .422** | .446** | .023 | -.091 | .599** | .200* | 1.000 | .131 |
| PANSS | -.083 | -.146 | -.032 | .055 | -.083 | .076 | .075 | .065 | -.181 | -.031 | .049 | .112 | .131 | 1.000 |

* p<0.05 **p<0.01

BMI: body mass index, FG: fasting glucose, TG: triglyceride, TC: total cholesterol, LDL: low density lipoprotein, HDL: high density lipoprotein.

Supplement Table 2 the Spearman relation test in drug naïve participants

| Drug naïve participants | Gender | Smoke | Course(y) | Olanzapine Concentration | weight | BMI | BMI rate | FG | TG | TC | LDL | HDL | PANSS |
| --- | --- | --- | --- | --- | --- | --- | --- | --- | --- | --- | --- | --- | --- |
| Gender | 1.000 | 0.030 | 0.114 | -0.103 | 0.086 | 0.148 | 0.194 | -0.042 | -0.248 | -0.080 | 0.092 | .313^*^ | 0.108 |
| Smoke | 0.030 | 1.000 | -0.035 | 0.314^*^ | 0.256 | 0.275 | 0.228 | .311^*^ | -0.275 | 0.007 | 0.026 | -0.055 | -0.064 |
| Course(y) | 0.114 | -0.035 | 1.000 | -0.014 | 0.021 | 0.041 | 0.057 | 0.061 | -0.167 | 0.023 | 0.035 | -0.030 | -0.149 |
| Olanzapine Concentration | -0.103 | .314^*^ | -0.014 | 1.000 | .375^**^ | .365^**^ | .324^*^ | 0.105 | 0.120 | .313^*^ | .472^**^ | 0.145 | 0.118 |
| Weight | 0.086 | 0.256 | 0.021 | 0.375^**^ | 1.000 | .987^**^ | .977^**^ | 0.096 | -0.095 | 0.114 | 0.137 | .377^**^ | 0.126 |
| BMI | 0.148 | 0.275 | 0.041 | 0.365^**^ | .987^**^ | 1.000 | .988^**^ | 0.087 | -0.137 | 0.104 | 0.147 | .388^**^ | 0.112 |
| BMI rate | 0.194 | 0.228 | 0.057 | 0.324^*^ | .977^**^ | .988^**^ | 1.000 | 0.019 | -0.155 | 0.090 | 0.164 | .435^**^ | 0.151 |
| FG | -0.042 | .311^*^ | 0.061 | 0.105 | 0.096 | 0.087 | 0.019 | 1.000 | -0.164 | 0.156 | -0.025 | -0.120 | -0.290 |
| TG | -0.248 | -0.275 | -0.167 | 0.120 | -0.095 | -0.137 | -0.155 | -0.164 | 1.000 | 0.038 | 0.132 | -0.050 | -0.085 |
| TC | -0.080 | 0.007 | 0.023 | 0.313^*^ | 0.114 | 0.104 | 0.090 | 0.156 | 0.038 | 1.000 | .610^**^ | .482^**^ | -0.015 |
| LDL | 0.092 | 0.026 | 0.035 | 0.472^**^ | 0.137 | 0.147 | 0.164 | -0.025 | 0.132 | .610^**^ | 1.000 | .485^**^ | 0.115 |
| HDL | .313^*^ | -0.055 | -0.030 | 0.145 | .377^**^ | .388^**^ | .435^**^ | -0.120 | -0.050 | .482^**^ | .485^**^ | 1.000 | 0.221 |
| PANSS | 0.108 | -0.064 | -0.149 | 0.118 | 0.126 | 0.112 | 0.151 | -0.290 | -0.085 | -0.015 | 0.115 | 0.221 | 1.000 |

* p<0.05 **p<0.01

Supplement Table 3 the Spearman relation test in Chronic participants

|  | Gender | Smoke | Metabolic Risk | Course(y) | Olanzapine Concentration | weight | BMI | BMI rate | FG | TG | TC | LDL | HDL | PANSS |
| --- | --- | --- | --- | --- | --- | --- | --- | --- | --- | --- | --- | --- | --- | --- |
| Gender | 1.000 | -0.011 | 0.072 | -0.202 | 0.231 | 0.024 | 0.073 | 0.068 | 0.101 | -0.012 | 0.089 | 0.039 | -0.069 | -0.165 |
| Smoke | -0.011 | 1.000 | 0.112 | 0.054 | 0.185 | -0.108 | -0.102 | -0.132 | 0.059 | -0.157 | 0.204 | 0.021 | 0.027 | -.290* |
| Metabolic Risk | 0.072 | 0.112 | 1.000 | -0.155 | 0.020 | -.313* | -.315* | -.282* | 0.103 | -0.125 | 0.014 | 0.034 | -0.050 | -.267* |
| Course(y) | -0.202 | 0.054 | -0.155 | 1.000 | -.267* | 0.052 | 0.048 | 0.016 | -0.095 | -0.172 | -0.073 | -0.079 | 0.016 | 0.170 |
| Olanzapine Concentration | 0.231 | 0.185 | 0.020 | -.267* | 1.000 | -0.208 | -0.210 | -0.204 | 0.225 | -0.109 | -0.210 | 0.142 | -0.238 | -.294* |
| Weight | 0.024 | -0.108 | -.313* | 0.052 | -0.208 | 1.000 | .992** | .988** | -0.012 | 0.071 | .284* | 0.081 | .399** | 0.075 |
| BMI | 0.073 | -0.102 | -.315* | 0.048 | -0.210 | .992** | 1.000 | .990** | -0.005 | 0.077 | .281* | 0.099 | .406** | 0.075 |
| BMI rate | 0.068 | -0.132 | -.282* | 0.016 | -0.204 | .988** | .990** | 1.000 | -0.004 | 0.065 | .282* | 0.093 | .432** | 0.053 |
| FG | 0.101 | 0.059 | 0.103 | -0.095 | 0.225 | -0.012 | -0.005 | -0.004 | 1.000 | .282* | 0.246 | 0.107 | 0.089 | -0.107 |
| TG | -0.012 | -0.157 | -0.125 | -0.172 | -0.109 | 0.071 | 0.077 | 0.065 | .282* | 1.000 | 0.220 | 0.152 | -0.066 | 0.028 |
| TC | 0.089 | 0.204 | 0.014 | -0.073 | -0.210 | .284* | .281* | .282* | 0.246 | 0.220 | 1.000 | 0.221 | .642** | 0.092 |
| LDL | 0.039 | 0.021 | 0.034 | -0.079 | 0.142 | 0.081 | 0.099 | 0.093 | 0.107 | 0.152 | 0.221 | 1.000 | 0.100 | 0.063 |
| HDL | -0.069 | 0.027 | -0.050 | 0.016 | -0.238 | .399** | .406** | .432** | 0.089 | -0.066 | .642** | 0.100 | 1.000 | 0.070 |
| PANSS | -0.165 | -.290* | -.267* | 0.170 | -.294* | 0.075 | 0.075 | 0.053 | -0.107 | 0.028 | 0.092 | 0.063 | 0.070 | 1.000 |

* p<0.05 **p<0.01

Supplement Table 4 the Spearman relation test in Metabolic Risk- participants

|  | Gender | Course(y) | Olanzapine Concentration | weight | BMI | BMI rate | FG | TG | TC | LDL | HDL | PANSS |
| --- | --- | --- | --- | --- | --- | --- | --- | --- | --- | --- | --- | --- |
| Gender | 1.000 | -0.088 | -0.269 | .127 | .212 | .193 | 0.000 | .058 | .423 | -.260 | .063 | .411 |
| Course(y) | -0.088 | 1.000 | -0.631^**^ | .138 | .137 | .117 | -.312 | -.404 | -.118 | -.323 | .192 | .641^**^ |
| Olanzapine Concentration | -0.269 | -0.631^**^ | 1.000 | .003 | -.033 | .021 | .447 | .472^*^ | -.172 | .492^*^ | -.318 | -.643^**^ |
| Weight | 0.127 | 0.138 | 0.003 | 1.000 | .990^**^ | .987^**^ | .327 | -.149 | .495^*^ | .354 | .566^*^ | .175 |
| BMI | 0.212 | 0.137 | -0.033 | .990^**^ | 1.000 | .987^**^ | .302 | -.101 | .539^*^ | .360 | .549^*^ | .225 |
| BMI rate | 0.193 | 0.117 | 0.021 | .987^**^ | .987^**^ | 1.000 | .297 | -.165 | .525^*^ | .352 | .566^*^ | .241 |
| FG | 0.000 | -0.312 | 0.531^*^ | .327 | .302 | .297 | 1.000 | .382 | .314 | .353 | .364 | -.258 |
| TG | 0.058 | -0.404 | 0.472^*^ | -.149 | -.101 | -.165 | .382 | 1.000 | .156 | .488^*^ | -.307 | -.336 |
| TC | 0.423 | -0.118 | -0.172 | .495^*^ | .539^*^ | .525^*^ | .314 | .156 | 1.000 | .128 | .800^**^ | -.001 |
| LDL | -0.260 | -0.323 | 0.492^*^ | .354 | .360 | .352 | .353 | .488^*^ | .128 | 1.000 | .086 | -.186 |
| HDL | 0.063 | 0.192 | -0.318 | .566^*^ | .549^*^ | .566^*^ | .364 | -.307 | .800^**^ | .086 | 1.000 | .030 |
| PANSS | 0.411 | 0.641^**^ | -0.643^**^ | .175 | .225 | .241 | -.258 | -.336 | -.001 | -.186 | .030 | 1.000 |

* p<0.05 **p<0.01

Supplement Table 5 the Spearman relation test in Metabolic Risk+ participants

|  | Gender | Course(y) | Olanzapine Concentration | weight | BMI | BMI rate | FG | TG | TC | LDL | HDL | PANSS |
| --- | --- | --- | --- | --- | --- | --- | --- | --- | --- | --- | --- | --- |
| Gender | 1.000 | -.229 | .448** | .008 | .044 | .038 | .151 | -.020 | -.087 | .166 | -.126 | -.303 |
| Course(y) | -.229 | 1.000 | -.138 | -.076 | -.080 | -.086 | .025 | -.119 | -.034 | .014 | -.065 | .010 |
| Olanzapine Concentration | .448** | -.138 | 1.000 | -.273 | -.263 | -.271 | .086 | -.312* | -.221 | .016 | -.195 | -.156 |
| Weight | .008 | -.076 | -.273 | 1.000 | .991** | .988** | -.143 | .103 | .217 | .007 | .340* | -.086 |
| BMI | .044 | -.080 | -.263 | .991** | 1.000 | .992** | -.115 | .104 | .197 | .026 | .365* | -.093 |
| BMI rate | .038 | -.086 | -.271 | .988** | .992** | 1.000 | -.128 | .083 | .202 | .013 | .391* | -.092 |
| FG | .151 | .025 | .086 | -.143 | -.115 | -.128 | 1.000 | .275 | .163 | -.018 | -.053 | -.001 |
| TG | -.020 | -.119 | -.312* | .103 | .104 | .083 | .275 | 1.000 | .228 | .044 | -.026 | .056 |
| TC | -.087 | -.034 | -.221 | .217 | .197 | .202 | .163 | .228 | 1.000 | .242 | .616** | .117 |
| LDL | .166 | .014 | .016 | .007 | .026 | .013 | -.018 | .044 | .242 | 1.000 | .110 | .077 |
| HDL | -.126 | -.065 | -.195 | .340* | .365* | .391* | -.053 | -.026 | .616** | .110 | 1.000 | .045 |
| PANSS | -.303 | .010 | -.156 | -.086 | -.093 | -.092 | -.001 | .056 | .117 | .077 | .045 | 1.000 |

* p<0.05 **p<0.01

Supplement table 6 three group analysis of the baseline demographic and clinical data

|  | **Drug-naïve group (n=51)** |  | **Risk^+^ group (n=47)** |  | **Risk^-^ group (n=19)** |  | **p-value** | **D vs R^+^** | **D vs R^-^** | **R^+^ vs R^-^** |
| --- | --- | --- | --- | --- | --- | --- | --- | --- | --- | --- |
| **Demographic Statistics** |  |  |  |  |  |  |  |  |  |  |
| Gender (male, female) | (19, 32) |  | (21, 26) |  | (10, 9) |  | 0.482 |  |  |  |
| Age (y) | (24.83 30.17) |  | (35.74 42.22) |  | (33.54 43.30) |  | <0.001 | .000 | .000 | .841 |
| Smoking (yes, no) | (6, 45) |  | (2, 45) |  | (0, 19) |  | 0.219 |  |  |  |
| **Clinical Statistics** |  |  |  |  |  |  |  |  |  |  |
| Course | (0.60 0.73) |  | (8.84 12.01) |  | (9.12 14.35) |  | <0.000 | .000 | .000 | .236 |
| Olanzapine dose | (17.93 19.32) |  | (18.23 20.17) |  | (17.94 19.96) |  | 0.594 | .310 | .670 | .737 |
| Olanzapine Concentration | (55.18 71.10) |  | (63.77 87.56) |  | (53.81 92.17) |  | 0.200 | .083 | .298 | .780 |
| Height | (1.61 1.66) |  | (1.62 1.67) |  | (1.61 1.66) |  | 0.832 | .545 | .826 | .816 |
| Weight | (53.29 58.54) |  | (58.05 65.57) |  | (58.48 70.89) |  | 0.006 | .012 | .005 | .357 |
| BMI | (20.25 21.46) |  | (21.69 23.82) |  | (22.01 26.09) |  | 0.000 | .004 | .000 | .138 |
| Fasting Glucose | (4.41 4.71) |  | (4.75 5.10) |  | (4.65 5.35) |  | 0.003 | .003 | .007 | .649 |
| Triglyceride | (1.01 1.46) |  | (1.06 1.29) |  | (1.12 1.57) |  | 0.594 | .633 | .504 | .310 |
| Total Cholesterol | (3.74 4.24) |  | (4.52 5.13) |  | (4.32 5.24) |  | 0.000 | .000 | .003 | .851 |
| LDL | (2.14 2.51) |  | (1.73 2.13) |  | (1.93 2.60) |  | 0.014 | .005 | .764 | .068 |
| HDL | (1.14 1.29) |  | (1.14 1.33) |  | (1.04 1.34) |  | 0.839 | .729 | .744 | .566 |
| PANSS | (79.97 87.87) |  | (86.43 93.57) |  | (85.94 93.12) |  | 0.041 | .017 | .095 | .888 |

D: drug-naïve group, R^+^: Metabolic Risk^+^ group, R^-^: Metabolic Risk^–^ group.

Supplement table 7 Subgroup analysis of change of clinical outcomes between baseline and endpoint ^a^

|  | **Metabolic Risk^+^ group (n= 47)** | | | | **Metabolic Risk^-^ group (n= 19)** | | | | **Repeated Measurement** | | **Chang of Outcomes** | |
| --- | --- | --- | --- | --- | --- | --- | --- | --- | --- | --- | --- | --- |
|  | Week 0 | Week 8 | Δ | p-value | Week 0 | Week 8 | Δ | p-value | **F** | **P value** | p-value | p-adj |
| Weight | 61.80 (58.05 65.57) | 61.21 (57.83 64.60) | -0.60 (-1.68 0.49) | 0.813 | 64.68 (58.48 70.89) | 66.79 (60.16 73.41) | 2.11 (0.62 3.59) | 0.629 | 1.575 | 0.214 | 0.007 | 0.007 |
| BMI | 22.76 (21.69 23.82) | 22.56 (21.63 23.49) | -0.19 (-0.61 0.22) | 0.785 | 24.05 (22.01 26.09) | 24.85 (22.61 27.10) | 0.80 (0.24 1.37) | 0.581 | 3.226 | 0.077 | 0.008 | 0.008 |
| Fasting Glucose | 4.93 (4.75 5.10) | 5.14 (4.88 5.39) | 0.22 (-0.02 0.45) | 0.175 | 5.00 (4.65 5.35) | 4.98 (4.61 5.35) | -0.02 (-0.44 0.40) | 0.941 | 0.049 | 0.825 | 0.295 | 0.408 |
| Triglyceride | 1.18 (1.06 1.29) | 2.15 (1.74 2.56) | 0.96 (0.56 1.37) | <0.001 | 1.35 (1.12 1.57) | 2.30 (1.81 2.79) | 0.96 (0.58 1.34) | 0.001 | 0.575 | 0.451 | 0.990 | 0.972 |
| Total Cholesterol | 4.83 (4.52 5.13) | 5.09 (4.79 5.38) | 0.26 (-0.02 0.53) | 0.224 | 4.78 (4.32 5.24) | 5.06 (4.37 5.75) | 0.28 (-0.26 0.82) | 0.483 | 0.021 | 0.885 | 0.933 | 0.756 |
| LDL | 1.93 (1.73 2.13) | 2.55 (2.31 2.78) | 0.62 (0.38 0.85) | <0.001 | 2.27 (1.93 2.60) | 2.79 (2.34 3.24) | 0.52 (0.19 0.86) | 0.059 | 0.188 | 0.666 | 0.666 | 0.659 |
| HDL | 1.24 (1.14 1.33) | 1.13 (1.02 1.23) | -0.08 (-0.18 0.02) | 0.122 | 1.19 (1.04 1.34) | 1.14 (0.93 1.35) | -0.01 (-0.21 0.20) | 0.686 | 0.035 | 0.853 | 0.455 | 0.405 |
| PANSS | 90.00 (86.43 93.57) | 59.46 (55.89 63.03) | -30.83 (-35.58 -26.08) | <0.001 | 89.53 (85.94 93.12) | 65.75 (61.45 70.05) | -22.88 (-28.21 -17.54) | <0.001 | 0.830 | 0.366 | 0.056 | 0.115 |

^a^ To compare baseline and endpoint within each group, ANOVA was conducted for continues variables. Mann-Whitney test was conducted if not normal distributed. To compare the change of outcomes between two groups, the General linear regression random effect model with course, smoking, age adjusted. BMI: body mass index. Repeated Measurement analysis was conducted to compare the change of outcomes between two groups over time.

LDL: low density lipoprotein. HDL: high density lipoprotein. PANSS: positive and negative symptom scale.

Supplement table 8 the Spearman relation test of change of weight and BMI

| All participants | Baseline weight | Baseline BMI | age | Course of disease | Medication history | PANSS |
| --- | --- | --- | --- | --- | --- | --- |
| Change of weight | -.285** | -.251** | -.209* | -.356** | -.313* | -.232* |
| Change of BMI | -.323** | -.258** | -.211* | -.340** | -.315* | -.222* |
| Change of TG | .113 | .106 | .119 | .073 | -.125 | .162 |

* p<0.05 **p<0.01 TG: triglyceride

Supplement table 9 the Multiple Linear regression analysis of change of weight and BMI

|  | Baseline weight/BMI | | age | | Course of disease | | Medication history | | PANSS | | Model Summary | |
| --- | --- | --- | --- | --- | --- | --- | --- | --- | --- | --- | --- | --- |
|  | β | p | β | p | β | p | β | p | β | p | Adjusted R Square | P-value |
| Change of weight | -0.222 | 0.015 | -0.029 | 0.782 | 0.070 | 0.594 | -0.406 | 0.001 | -0.037 | 0.674 | 0.196 | <0.001 |
| Change of BMI | -0.231 | 0.015 | -0.047 | 0.654 | 0.093 | 0.493 | -0.391 | 0.001 | -0.044 | 0.615 | 0.187 | <0.001 |

Supplement table 10 the Spearman relation test of change of triglyceride

| All participants | Baseline fasting Glucose | Baseline Cholesterol |
| --- | --- | --- |
| Change of TG | .218* | .199* |

* p<0.05 **p<0.01 TG: triglyceride

Supplement table 11 the Multiple Linear regression analysis of change of triglyceride

|  | Cholesterol | | Glucose | | Medication history | | Model Summary | |
| --- | --- | --- | --- | --- | --- | --- | --- | --- |
|  | β | p | β | p | β | p | Adjusted R Square | P-value |
| Change of TG | 0.108 | 0.288 | -0.010 | 0.915 | 0.200 | 0.055 | 0.04 | 0.058 |
