# Supplementary material for: Association between olanzapine concentration and metabolic dysfunction in drug-naive and chronic patients: similarities and differences
Source: Schizophrenia (Heidelb). 2022 Feb 28;8(1):9. doi: 10.1038/s41537-022-00211-5 (PMC8885747; doi:10.1038/s41537-022-00211-5)
Supplement: Supplementary file 2 — Supplement Figure [file 41537_2022_211_MOESM2_ESM.doc]

**Study Flow Diagram**

**Allocation**

**Analysis**

**Follow-Up**

**Enrollment**

Assessed for eligibility (n= 120)

Excluded (n=3)

♦  Not meeting inclusion criteria (n=2)

♦  Declined to participate (n=1)

Analysed (n= 51 )

Lost to follow-up (n= 0 )

Discontinued intervention (n= 0 )

Drug naïve patients (n=51)

♦ Received olanzapine therapy (n= 51 )

Lost to follow-up (n= 0 )

Discontinued intervention (n= 0 )

Chronic Patients (n=66)

♦ Received olanzapine therapy (n= 66 )

Analysed (n= 66 )

Assigned (n=117)
